# Transcriptomics Reveals the Molecular Basis for Methyl Jasmonate to Promote the Synthesis of Monoterpenoids in *Schizonepeta tenuifolia* Briq.

**DOI:** 10.3390/cimb45040179

**Published:** 2023-03-24

**Authors:** Jianling Shi, Yingjing Cui, Jimeng Zhang, Liqiong Sun, Xiaoqing Tang

**Affiliations:** Institute of Medicinal Materials, Nanjing Agricultural University, Nanjing 210095, China

**Keywords:** *Schizonepeta tenuifolia*, methyl jasmonate, transcriptome, monoterpenoids, pulegone

## Abstract

Background: Methyl jasmonate has an important effect on the synthesis of plant secondary metabolites. *Schizonepeta tenuifolia* Briq. has a wide range of pharmacological effects and the secondary metabolites are dominated by monoterpenes (pulegone, menthone). Objective: It is essential to determine the changes in secondary metabolites in *S. tenuifolia* under methyl jasmonate treatment and to probe the molecular mechanism. This can improve the accumulation of secondary metabolites in the medicinal plant *S. tenuifolia* and enrich the information gene expression at different MeJA levels, which can help to elucidate the molecular mechanism of monoterpenoid synthesis in *S. tenuifolia*. Methods: In this study, we determined the changes in the content of monoterpenoids in *S. tenuifolia* under methyl jasmonate treatment. Meanwhile, we established a transcriptome database of *S. tenuifolia* under methyl jasmonate level using high-throughput sequencing. Results: A certain concentration of MeJA promoted the accumulation of monoterpenoids in *S. tenuifolia*. The transcriptome database of *S. tenuifolia* leaves under 0, 50, 100 and 250 μM MeJA treatment was established. We generated 88,373 unigenes with an N50 length of 2678 bp, of which 50,843 (57.53%) can be annotated in at least one database. Compared with the CK (0 μM) group, 12,557 (50 μM), 15,409 (100 μM) and 13,286 (250 μM) differentially expressed genes were identified. GO and KEGG enrichment analysis revealed that JA signal transduction and monoterpenoid synthesis were the two most significant enrichment pathways. The expression levels of related DEGs involved in JA signaling and monoterpenoid synthesis were significantly up-regulated by MeJA. In addition, our phenotypic and differentially expressed gene association analysis revealed that monoterpenoid biosynthesis in *S. tenuifolia* was more associated with genes involved in plant trichome branching, phytohormone signaling and transcriptional regulation. Conclusions: This study confirmed that methyl jasmonate significantly promoted monoterpenoid biosynthesis in *S. tenuifolia*. A large number of genes responding to methyl jasmonate were associated with JA signaling and monoterpenoid biosynthesis.

## 1. Introduction

*Schizonepeta tenuifolia* Briq. (Jingjie in Chinese) is an annual herb in the labiaceae family and is a very well-known medicinal plant with a long history of use in China, Korea and Japan. The dried above-ground parts of *S. tenuifolia* can be used as an herb, as well as in drinks, teas and recipes. Additionally, they have a wide range of pharmacological effects, namely antibacterial, antioxidant, antipyretic and immunomodulatory [1,2,3]. The above-ground parts are rich in volatile oils, of which the most important components are monoterpenoids, including pulegone and menthone [4]. Studies have shown that pulegone can be used as a good antioxidant, antibacterial, and antiviral agent [5], and also as a good anti-anxiety drug [6]. Some menthone derivatives also have analgesic and anticonvulsant effects [7]. However, the biosynthetic mechanism of pulegone has only been extensively studied in peppermint (*Mentha piperita* L.) [8,9] and spearmint (*Mentha spicata* L.) [10], and the mechanism of synthesis in *S. tenuifolia* is still unclear. Only three key genes related to pulegone biosynthesis, including *DXS* [11], *LS* [12] and *L3OH* [13], have been cloned in *S. tenuifolia*.

As a “wound hormone”, methyl jasmonate plays an important role in plant response to biotic and abiotic stresses and is a key regulator in inducing the synthesis of secondary metabolites in plants [14,15,16]. Studies have shown that MeJA regulates the synthesis of various secondary metabolites of medicinal plants, such as artemisinin, ginsenoside and vinblastine [17,18,19,20]. Chen et al. found that MeJA promoted the release of volatile substances in *Styrax japonicus* [21]. In a newly published study on paclitaxel biosynthesis, MeJA was found to induce the accumulation of paclitaxel in taxus cultures [22]. Transcription factors play an important role in the regulation of plant metabolism by MeJA, and are an indispensable member of plant growth and development, stress response and metabolite biosynthesis [23,24,25,26], such as the transcription factor MYC4 positively regulates MeJA-induced terpenoid synthesis in lavender [27]. However, there are few studies on the mechanism of JA signal transduction and monoterpenoid biosynthesis regulated by MeJA in *S. tenuifolia*.

In our previous study, we found that the density of glandular trichomes in the upper and lower epidermis of *S. tenuifolia* leaves increased under MeJA treatment, and a significant increase in the content of secondary metabolites such as flavonoids, phenols and monoterpenoids occurred. We also evaluated the antioxidant capacity and found that the antioxidant capacity of *S. tenuifolia* leaves was significantly enhanced under MeJA treatment. However, we do not know the molecular mechanisms involved, and in view of this, we believe that it is urgent to reveal the regulatory mechanisms of MeJA on secondary metabolites of *S. tenuifolia* [28]. Transcriptome sequencing is an accurate and efficient sequencing method that has grown to be one of the most popular histological assays. Because it allows unbiased quantification of the entire genome and identification of differentially expressed genes, it has been used to reveal the mechanisms of plant response to various biotic and abiotic stresses at the molecular level [29,30,31,32]. Therefore, in this study, transcriptome sequencing was used to analyze the differential gene expression transcription profiles of *S. tenuifolia* under different MeJA levels, to identify gene clusters in response to MeJA in *S. tenuifolia*, and to screen genes and transcription factors involved in JA signal transduction and monoterpenoid biosynthesis. The present study helps us to further investigate the molecular mechanism of MeJA-induced monoterpenoid biosynthesis in *S. tenuifolia* and facilitate the study of the biosynthesis mechanism of pulegone.

## 2. Materials and Methods

### 2.1. Plant Materials and Treatments

The large and plump seeds of *S. tenuifolia* were sown in a plastic basin containing the cultivation substrate, and cultured in a greenhouse (118.51′ E; 32.1′ N) of Nanjing Agricultural University, Nanjing, China. The greenhouse conditions were natural light, 30 °C in the day, 25 °C at night, and 50–70% humidity. MeJA treatment was started at the flowering stage (50 days after emergence). MeJA (M8640, Solarbio, Beijing, China) was dissolved in a small amount of ethanol (5mL) and then diluted with H_2_O to give the desired concentrations (50 μmol·L^−1^, 100 μmol·L^−1^, 250 μmol·L^−1^). The leaves were carefully sprayed with 50 μM, 100 μM and 250 μM MeJA, and the control treatment was sprayed with distilled water containing the same ethanol water solution. The leaves were harvested from 24 h after treatment, immediately frozen in liquid nitrogen, and stored at −80 °C. The treatment was repeated at least three times in each group.

### 2.2. Determination of Pulegone and Menthone Content by GC-MS

Leaves of *S. tenuifolia* at flowering stage were collected, and 7 g fresh leaves were accurately weighed and placed in a round-bottom flask with 100 mL deionized water for distillation. Camphor was used as the internal standard (final concentration at 15 ng·μL^−1^), and n-hexane as the carrier solvent [33].

Detection was performed using a triple quadrupole gas chromatography-mass spectrometer (Trace1310/TSQ 9000, Thermo Scientific). Analytical conditions: TG-5 ms column (0. 25 mm × 30 m, 0. 25 μm); programmed ramp-up: from 50 °C, hold for 5 min, ramp-up to 90 °C at 10 °C·min^−1^, hold for 10 min, ramp-up to 160 °C at 10 °C·min^−1^, hold for 10 min, ramp-up to 250 °C at 10 °C·min^−1^, hold for 10 min. The carrier gas was helium at a flow rate of 1.00 mL·min^−1^, a splitting ratio of 40:1, and an injection volume of 1.0 μL. MS conditions: EI ion source, ion source temperature 230 °C, interface temperature 250 °C, electron energy 70 eV, scanning range 30–1000 m·z^−1^. Components were identified according to the NIST17 mass spectrometry standard database.

### 2.3. RNA Extraction and cDNA Library Construction

Total RNA was isolated using the SV Total RNA Isolation System kit (Promega, Beijing, China). RNA concentration and purity were measured using NanoDrop2000 (Thermo Fisher Scientific, Wilmington, DE, USA). RNA integrity was assessed by the RNA Nano 6000 Assay Kit of the Agilent Bioanalyzer 2100 system (Agilent Technologies, Santa Clara, CA, USA).

Sequencing libraries were generated using NEBNextR UltraTM Directional RNA Library Prep Kit for IlluminaR (NEB, USA) following manufacturer’s recommendations and index codes were added to attribute sequences to each sample. Magnetic beads with Oligo (dT) were used to enrich mRNA, and which was randomly interrupted by Fragmentation Buffer. The first cDNA strand was synthesized with six-base random primers using mRNA as template and the buffer, dNTPs, RNase H and DNA polymerase I were added to synthesize the second cDNA strand. The cDNA was purified using AMPure XP beads, and the purified double-stranded cDNA was then end-repaired, A-tailed and ligated to sequencing junctions, followed by fragment size selection using AMPure XP beads, and cDNA libraries were obtained by PCR enrichment. Finally, library quality was assessed using the Agilent Bioanalyzer 2100 system (Agilent Technology, California, USA).

### 2.4. Transcriptome Sequencing and Assembly

The clustering of the index-coded samples was performed on a cBot Cluster Generation System by using TruSeq PE Cluster Kit v4-cBot-HS (Illumia, San Diego, CA, USA) according to the manufacturer’s instructions. After cluster generation, the library preparations were sequenced on an Illumina Hiseq Xten platform and paired-end reads were generated. Raw sequencing data files (51.09 GB) were uploaded to the NCBI Sequence Read Archive database (project number: PRJNA908244). 

The raw data obtained by high-throughput sequencing are quality-controlled and filtered to remove connectors and primer sequences, filter low quality bases and N-containing bases, and obtain high quality reads for subsequent analysis. FastQc (v0.11.8) [34] was used for quality control of clean data. The sequence assembly of the obtained high-quality clean data was performed using Trinity software (v2.4.0) to generate the transcript sequence of each gene, and the longest transcript in each gene was taken as unigene. 

### 2.5. Gene Functional Annotation

BLAST software (v2.2.26) [35] was used (screening parameter E-value ≤ 10–5) to compare Unigene sequences with NR [36], Swiss-Prot [37] GO [38], COG [39], KOG [40] and KEGG [41] databases, respectively. After predicting the amino acid sequence of unigene, HMMER [42] software was used to compare with Pfam [43] database to obtain annotation information of unigene. 

### 2.6. Differentially Expressed Gene (DEG) Analysis

Gene expression levels for each sample unigenes of each methyl jasmonate treatment were estimated by fragments per kilobase of transcript per million fragments mapped (FPKM). Differentially expressed gene (DEG) analysis between two groups (CK vs. MJ50, CK vs. MJ100, CK vs. MJ250) was completed by the DESeq2 and provided statistical routines, with a threshold of FDR (false discovery rate) < 0.05 and |log2 (Fold Change)|≥ 1 [44]. After screening for differential genes, GO and KEGG enrichment analysis of differential genes was performed according to the hypergeometric distribution principle.

### 2.7. Verification of Gene Expression Using Quantitative Real-Time PCR

Quantitative Real-Time PCR was used to verify the gene expression levels of each treatment group. We selected eight DEGs (DXS1, 902 bp; DXS3, 1491 bp; DXR, 1980 bp; GPS, 3257 bp; IPR, 1486 bp; JAZ3, 3527 bp; HMGR, 1610 bp; HMGS, 2068 bp) related to monoterpenoid synthesis and JA signaling in *S. tenuifolia* and primers were designed using Primer 5.0 software (Table S3). The *ACT* gene was used as an internal reference gene and *ACT* primers were extracted from the literature [13]. Total RNA was isolated using the RNAprep Pure Plant Plue Kit (TIANGEN BIOTECH, Beijing, China). 50–100 mg of MeJA-treated leaves of *S. tenuifolia* were taken and rapidly ground into powder in liquid nitrogen, and the subsequent operation was performed according to the kit instructions. The first strand cDNA was synthesized using TaKaRa PrimeScript™ RT reagent kit. 10 μL of reaction solution was prepared in RNase free centrifuge tubes, and the PCR reaction procedure was: 37 °C, 15 min; 85 °C, 5 s, and stored at 4 °C after the reaction. The qRT-PCR analysis was performed on QuantStudio 6 Flex using TSINGKE TSE202 2 × T5 Fast qPCR Mix (SYBR Green I) with three replications. The expression of each gene was calculated using 2^−ΔΔCT^.

## 3. Results

### 3.1. Quantitative Determination of Pulegone and Menthone Using GC-MS

**Figure 1:**
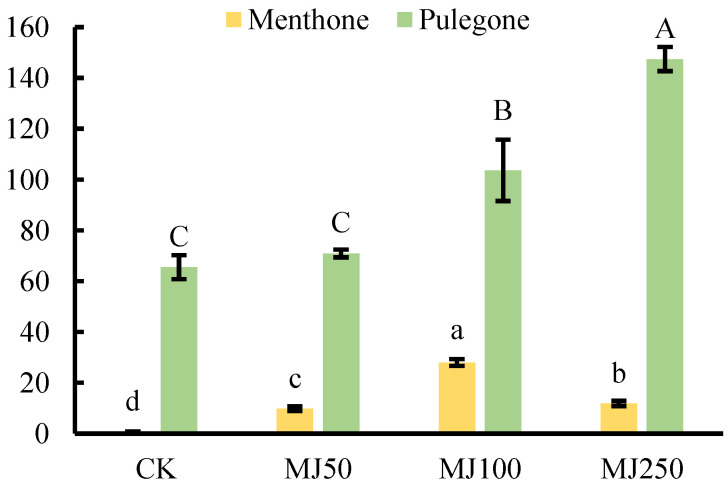
Variation of monoterpenoid content in *Schizonepeta tenuifolia* at different MeJA Levels. The uppercase letters and lowercase letters, respectively, represent the significant difference (*p* < 0.05) between the treatment groups of pulegone and menthone. CK 0 μM; MJ50 50 μM; MJ100 100 μM; MJ250 250 μM.

We used a GC-MS approach to examine the content of pulegone and menthone in plants with different MeJA concentrations, namely, 0 μM (CK), 50 μM (MJ50), 100μM (MJ100) and 250 μM (MJ250). The results indicated that exogenous MeJA promoted the accumulation of monoterpenoids in the leaves of *S. tenuifolia* (Figure 1). Pulegone content increased with the increase in MeJA concentration, and the highest accumulation amount was 147.48 ng·μL^−1^ at MJ250. However, the menthone content showed a downward trend, with the highest accumulation of 27.98 ng·μL^−1^ at MJ100. 

### 3.2. Transcriptomic Sequencing and De Novo Assembly

Transcriptome sequencing was performed to obtain transcribed cDNA libraries for a total of 12 samples, including four treatments (CK, MJ50, MJ100, MJ250), with each treatment repeated three times. We obtained 94.39 Gb clean data after removing the connector, primer sequence, N base and some low-quality reads. Clean data of each sample reached 7.19 Gb or above, and the percentage of Q30 base was more than 92.19% (Table S1). These high-quality clean reads were assembled by Trinity software, resulting in a total of 247,118 transcripts with an N50 length of 3187 bp. In total, 88,373 unigenes were finally obtained with an N50 length of 2678 bp. Unigenes included 25,482 (28.83%) articles within 300–500 bp length range, 26,415 (29.89%) at 500–1000 bp, 15,654 (17.71%) at 1000–2000 bp and 20,822 (23.56%) ≥2000 bp (Table 1, Figure S1).

### 3.3. Analysis of Differentially Expressed Genes

**Figure 2:**
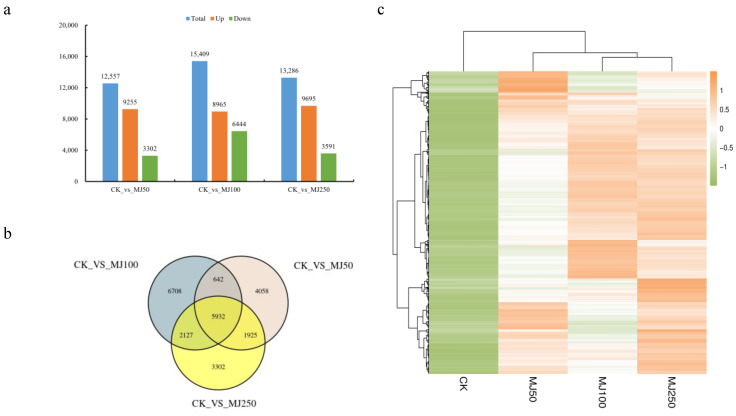
Analysis of differentially expressed genes in *S. tenuifolia* under different concentrations of MeJA treatment. (**a**) Statistics of DEGs between combinations; (**b**) Venn diagram of differentially expressed genes between combinations; (**c**) Heat map analysis of clustering of 5362 up−regulated DEGs expression pattern. CK: 0 μM, MJ50: 50 μM, MJ100: 100 μM, MJ250: 250 μM.

Using high-throughput RNA-sequencing, four gene libraries of 0 μM (CK), 50 μM (MJ50), 100 μM (MJ100) and 250 μM (MJ250) were established to screen differentially expressed genes between the MeJA-treated and unsprayed groups according to the expression level of each gene. We identified a total of 24,694 DEGs, including 12,557 for CK VS MJ50, 15,409 for CK VS MJ100, and 13,286 for CK VS MJ250 (Figure 2). We found the number of DEGs was the largest by comparing CK with MJ100, including 8965 up-regulated genes and 6444 down-regulated genes. However, there are the largest number of up-regulated genes between CK and MJ250 in each treatment group, which was 9695 genes. Additionally, compared with CK, the MJ50 condition had 9255 up-regulated and 3302 down-regulated DEGs. Meanwhile, there were 5362 up-regulated and 569 down-regulated DEG expressions after MeJA treatment (Figure S2), which indicated that some similar response mechanisms exist in plants in response to MeJA. In addition, we performed differential gene heat map analysis of 5362 up-regulated DEGs in both MJ50, MJ100 and MJ250 compared to CK (Figure 2c), which suggested unique gene expression in the MeJA treatment, indicating that the genes involved in transcriptional regulation varied under different MeJA conditions.

### 3.4. Gene Ontology Enrichment and KEGG Pathway Analysis of DEG

**Figure 3:**
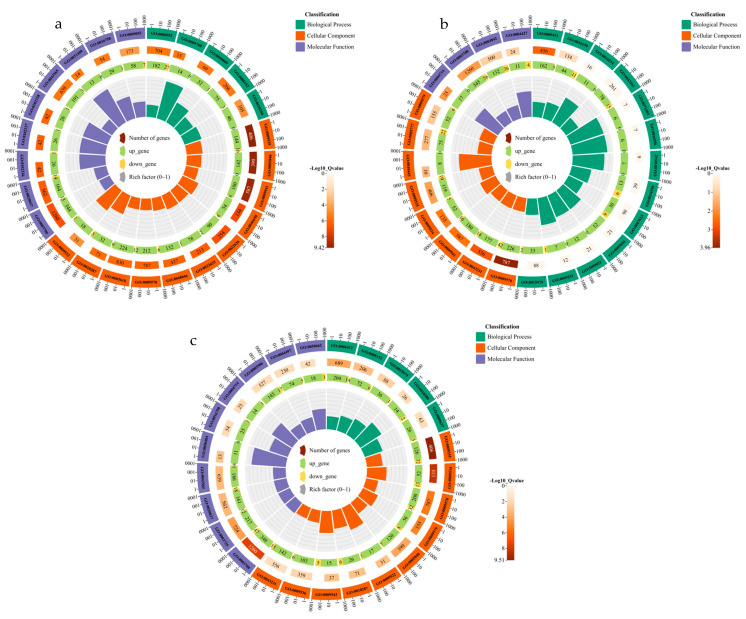
Enrichment analysis of the first 25 terms differentially expressed genes. (**a**) CK VS MJ50; (**b**) CK VS MJ100; (**c**) CK VS MJ250.

To further clarify the biological functions of DEGs, we performed GO (Top 25) enrichment analysis (Figure 3, Table S2). The results revealed that the most enriched term was transcription factor binding activity (GO:0003700), with 357, 401 and 395 DEGs annotated in CK VS MJ50, CK VS MJ100 and CK VS MJ250, respectively. Additionally, chloroplast stroma (GO:0009570) and chloroplast thylakoid membrane (GO:0009535) were also annotated in the three comparison groups. We found that large amounts of DEGs were annotated in the metabolic process (GO:0008152) term in both CK VS MJ50 and CK VS MJ250 comparison groups. Notably, a small number of DEGs were annotated to be associated with blue light uptake and response (GO:0071483, GO:0009637) in MJ100 and MJ250 compared to the CK group. Moreover, 93 DEGs of CK VS MJ100 were annotated in proteasome-mediated ubiquitin-dependent protein catabolic process (GO:0043161), and 12 DEGs were associated with regulation of the jasmonic acid mediated signaling pathway (GO:2000022).

**Figure 4:**
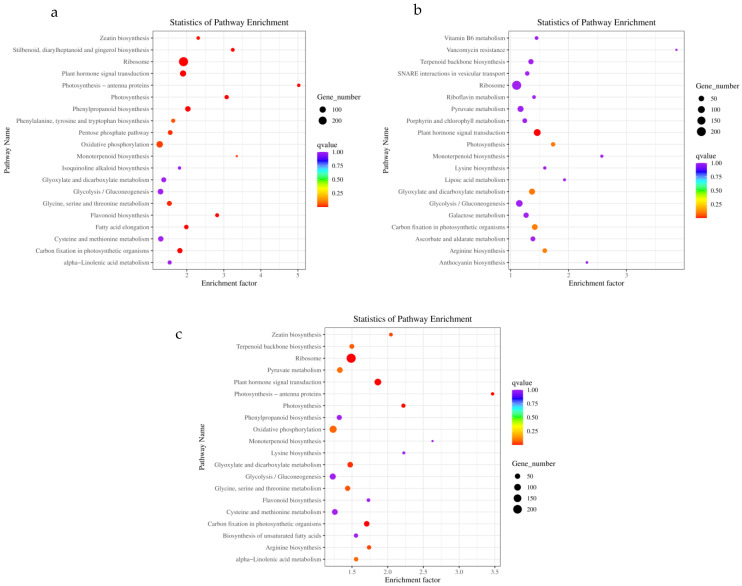
KEGG enrichment analysis of differential genes. (**a**) CK VS MJ50; (**b**) CK VS MJ100; (**c**) CK VS MJ250.

Based on the q-values and enrichment factor, we subjected the DEGs to KEGG enrichment analysis and screened 20 significantly enriched KEGG metabolic pathways (Figure 4). The results showed that the three comparison groups were significantly enriched in four terms of carbon metabolism, photosynthesis, plant hormone signal transduction and monoterpenoid biosynthesis, and most of them were up-regulated DEGs. In particular, the expression levels of the genes involved in monoterpenoid synthesis were up-regulated in all treatment groups, which further indicated that MeJA promoted the monoterpenoid synthesis of *S. tenuifolia*. Additionally, genes involved in terpenoid skeleton synthesis were significantly enriched in CK VS MJ100 and CK VS MJ250, suggesting that there were differences in the effects of different concentrations of MeJA on terpenoid synthesis in *S. tenuifolia*. In addition, some DEGs were enriched in CK VS MJ50 and CK VS MJ250 to correlate with flavonoid synthesis. 

### 3.5. Genes Involved in Jasmonate (JA) Signal Transduction-Related

**Figure 5:**
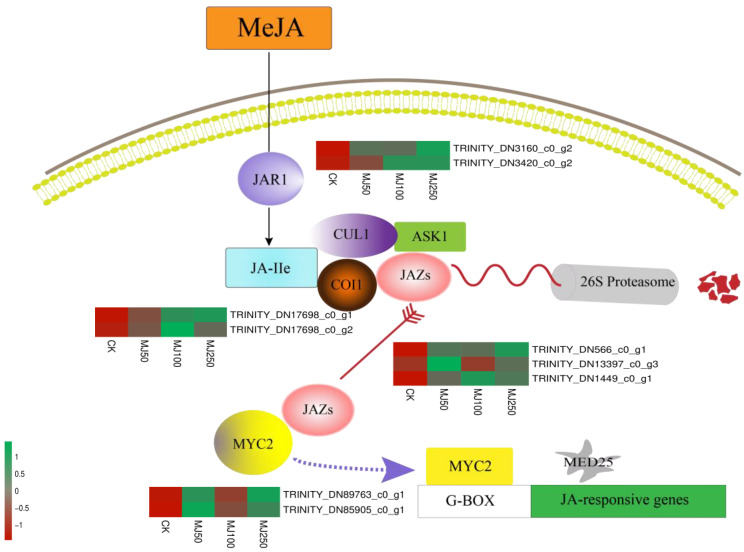
Expression patterns of genes involved in JA signaling. Each heat map from left to right: CK, MJ50, MJ100, MJ250. The color gradient from red to green represents transcript levels from low to high. JAR1 jasmonate−amido synthetase; COI1 coronatin−insensitive 1; JAZs Jasmonate ZIM domain (JAZ) proteins.

MeJA acts as a signaling molecule to which the plant organism can respond rapidly. In this study, the genes involved in JA signaling were identified and the expression patterns were analyzed under different concentrations of MeJA treatment (Figure 5). We identified two DEGs encoding jasmonate-amido synthetase (*JAR1*), their expression levels were up-regulated under MeJA treatment compared to the CK and reached the highest under MJ250 treatment with FPKM values of 3.97 (DN3160_c0_g2) and 5.39 (DN3420_c0_g2). The two DEGs encoding coronatin-insensitive 1 (*COI1*) had a similar expression pattern to *JAR1*, with expression levels upregulated in response to MeJA. Jasmonate ZIM domain (*JAZ*) proteins, as key regulators of jasmonate (JA) signaling, and the three encoded DEGs were all up-regulated genes. While the transcription factor MYC2 is the activator of downstream functional genes, the two encoded DEGs were expressed at different levels under different MeJA concentrations and were all up-regulated compared with the CK. It was worth noting that the expression level of MYC2 under MJ100 treatment was lower than that of the other two MeJA groups.

### 3.6. Prediction of Genes Related to Monoterpenoid Synthesis Pathway

**Figure 6:**
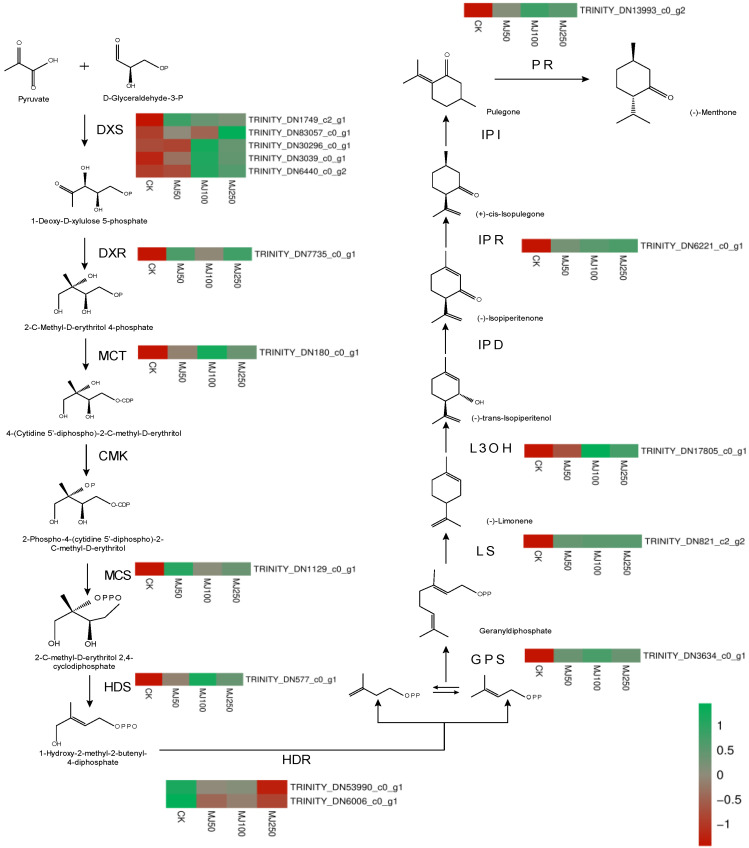
Monoterpenoid synthesis−related differentially expressed genes. Enzyme abbreviations: DXS 1−deoxy−D-xylulose−5−phosphate synthase; DXR 1−deoxy−D−xylulose−5−phosphate reductoisomerase; MCT 2−C−methyl−D−erythritol 4−phosphate cytidylyltransferase; MCS 2−C−methyl−D−erythritol 2,4−cyclodiphosphate synthase; HDS (E) −4−hydroxy-−3−methylbut−2−enyl−diphosphate synthase; HDR 4−hydroxy−3−methylbut−2−en−1−yl diphosphate reductase; GPS geranyl diphosphate synthase; LS (−)−limonene sythnase; L3OH Limonene−3−hydroxylase; IPR (−)−isopiperitenone redustase; PR pulegone reductase.

The monoterpenoids of *S. tenuifolia* are mainly based on pulegone and menthone, and pulegone is the precursors of menthone production. The monoterpenoid biosynthetic pathway of *S. tenuifolia* includes the synthesis of terpenoid skeleton and monoterpenoid skeleton modification (Figure 6). We annotated a total of 14 genes involved in terpenoid skeleton synthesis, including five 1-deoxy-D-xylulose-5-phosphate synthase (*DXS*) genes, of which four DEGs showed up-regulated expression levels under MeJA, whereas the remaining DEG (DN30296_c0_g1) showed down-regulated expression levels under MJ50 treatment compared with the CK group. The expression patterns of 2-C-methyl-D-erythritol 4-phosphate cytidylyltransferase (*MCT*) and (E)-4-hydroxy-3-methylbut-2-enyl-diphosphate synthase (*HDS*) were similar, and with the increase in MeJA concentration, they first increased and then decreased. However, although the expression levels of 1-deoxy-D-xylulose-5-phosphate reductoisomerase (*DXR*) and 2-C-methyl-D-erythritol 2,4-cyclodiphosphate synthase (*MCS*) were upregulated under the MeJA treatment compared with the CK, the expression levels in the MJ100 group were the lowest among the three treatment groups. Notably, the 4-hydroxy-3-methylbut-2-en-1-yl diphosphate reductase (*HDR*) gene was downregulated under the action of MeJA. 

The modification of the monoterpenoid skeleton relies on the action of various key enzymes. We identified a total of five genes involved in pulegone production, and encoding five key enzymes each. The gene expression level of geranyl diphosphate synthase (*GPS*), the first key enzyme for terpenoid skeleton modification, performed an increasing and then decreasing trend with MeJA concentration and reached a maximum under MJ100 treatment, with an FPKM value 5.41 times higher than that of CK. Limonene-3-hydroxylase (*L3OH*) expression levels were similar to *GPS* gene changes under MeJA. The genes for (-)-limonene sythnase (*LS*) and (-)-isopiperitenone reductase (*IPR*) had higher expression levels under MJ250 treatment. Pulegone reductase promotes the conversion of pulegone to menthone. Transcriptome data showed that the expression level of the gene encoding pulegone reductase (*PR*) reached the highest in MJ100, suggesting that *PR* may contribute to the production of menthone at the MJ100 level in *S. tenuifolia*.

### 3.7. Identification and Analysis of Transcription Factors

**Figure 7:**
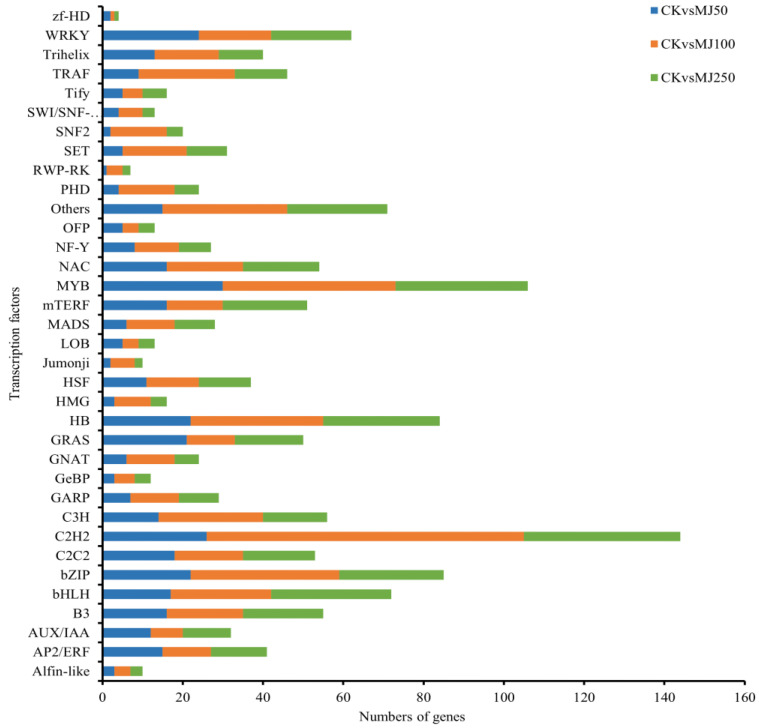
Identifcation and analysis of transcription factor gene expression levels in response to four MeJA treatments.

Transcription factors play an important role in plant growth and development, secondary metabolism and response to the external environment. Understanding the expression of transcription factors at different MeJA levels could provide a molecular basis for exploring the regulation of secondary metabolism in *S. tenuifolia*. A total of 887 differentially expressed transcription factors were identified from the four MeJA levels (Figure 7), of which the top five enriched transcription factor families were C2H2 (112), MYB (51), HB (43), bZIP (41) and bHLH (39). Analysis of the expression trend of the above transcription factors (Figure S3) showed that most of the transcription factors had high expression levels under MJ250 treatment, whereas they showed low expression levels in CK, MJ50 and MJ100. Most genes of MYB, HB, WRKY, bHLH and bZIP families were mostly up-regulated in expression level by MeJA, whereas C2H2 was mostly down-regulated. The GeBP family is involved in plant trichome development and growth, and we screened a total of five differentially expressed transcription factors of this family from transcriptome data, four of which were highly expressed at the MeJA level.

### 3.8. Weighted Gene Co-Expression Network (WGCNA) Analysis

**Figure 8:**
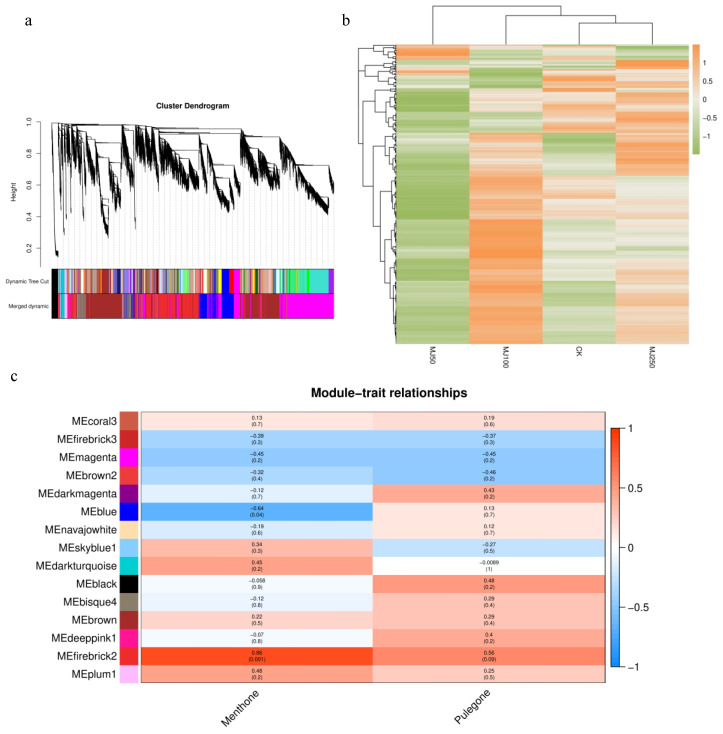
Weighted gene co-expression network analysis of the response of four MeJA−treated *S. tenuifolia* gene modules. (**a**) Gene hierarchy clustering dendrogram. Each color represents a module; (**b**) heat map analysis of ME firebrick 2 module gene clustering; (**c**) correlation analysis of gene modules with pulegone and menthone.

To further identify the gene expression patterns involved in the MeJA response, we performed weighted gene co-expression network analysis. Removing some low-level genes, 15 expression modules in total were obtained (Figure 8a). Correlating these modules with the phenotype of *S. tenuifolia*, we found that ME firebrick 2, containing 356 genes, was more correlated with pulegone and menthone (Figure 8b). Clustering heat map analysis of the genes contained in the above modules showed that most of the genes were expressed at higher levels under MJ100 treatment, and these genes were mainly related to trichome branching, systemic defense, signal transduction and regulation of transcription, which indicated that the molecular level and metabolic processes in plants respond to MeJA together. Moreover, MJ100 treatment may be more beneficial to the synthesis of monoterpenoids of *S. tenuifolia*. 

### 3.9. qRT-PCR Validation of Gene Expression Levels

**Figure 9:**
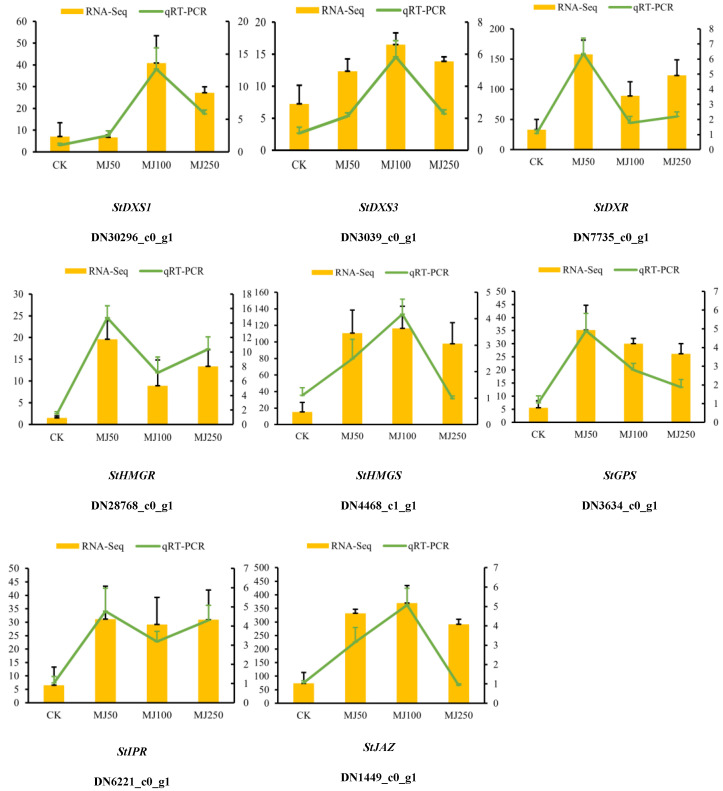
The qRT-PCR validation of eight differentially expressed genes. Bar graphs indicate RNA-Seq expression, solid lines indicate qRT-PCR relative expression. The left Y-axis is the FPKM value of RNA-Seq, and the right Y-axis is the qPCR relative expression value.

To verify the reliability of the transcriptomic data, we selected seven genes related to the synthesis of monoterpenoids and one gene related to JA signaling for qRT-PCR validation (Figure 9). The results showed that although there were some differences in expression ploidy, the expression pattern of the selected genes by qRT-PCR was generally consistent with the transcriptome data. The expression levels of all eight genes showed an increasing and then decreasing trend with increasing MeJA concentration. *StDXS1*, *StDXS3*, *StJAZ* and *StHMGS* had higher expression levels at MJ100, whereas the expression level of *StHMGR*, *StIPR*, *StGPS* and *StDXR* was MJ50. The qRT-PCR results demonstrated the reliability of the transcriptomic data, and thus the transcriptomic data could be useful for exploring the molecular mechanisms by which MeJA promotes the synthesis of monoterpenoids in *S. tenuifolia*.

## 4. Discussion

As a novel plant signaling molecule, MeJA not only regulates plant growth and development [45,46,47], but also acts as a regulator to promote the accumulation of plant secondary metabolites to resist external adversity stress [48,49,50]. In this study, the contents of volatile compounds (mainly pulegone and menthone) in *S. tenuifolia* were significantly increased under the action of MeJA, which is similar to previous studies in *Camellia sinensis*, *Castilleja tenuiflora* and *Lavandula angustifolia*. Jiang et al. found that MeJA improves tea quality by promoting terpenoid in tea, especially linalool, geraniol, and phenylethyl alcohol [51]. The substance basis of the medicinal value of *C. tenuiflora* is mainly terpenoid and phenolic substances. Studies showed that external application of MeJA promoted the synthesis of iridoid glycosides and phenolic compounds of *C. tenuiflora*, and also up-regulated the expression level of enzyme genes in its biosynthesis process [52]. In addition, Dong et al. showed that MeJA had a positive effect on the release of volatile compounds from the aromatic plant lavender [53]. 

We screened the differentially expressed genes treated with MeJA using high-throughput sequencing, and conducted GO and KEGG enrichment analysis. We found that the most significantly enriched term was transcriptional activation activity, indicating that MeJA may activate the binding of many transcription factors with downstream genes. Previous studies have shown that *PatDREB*, a transcription factor of the AP2/ERF family, activates the promoter of the patchoulol synthase gene in patchouli and positively regulates MeJA-induced synthesis of patchoulol [54]. Wang et al. found that DEGs with catalytic activity and binding activity accounted for the largest proportion in their study of the effect of MeJA on secondary metabolism and transcriptional profiles of *Lycoris aurea* [55]. Many researchers have found that JA is involved in the regulation of plant responses to light in species such as rice and arabidopsis, and JA has now been shown to be one of the key regulators of photosensitive pigment signal transduction [56]. Functional annotation of DEGs revealed that a few genes in *S. tenuifolia* were involved in the absorption and response of plants to blue light after MeJA treatment, so it was speculated that JA in *S. tenuifolia* might also be involved in the regulation of plant response to light. The expression of most key enzyme genes in the terpenoid and flavonoid synthesis pathway of *S. tenuifolia* was up-regulated by MeJA, leading to an increase in the content of related substances. The expression of 12 key enzyme genes for artemisinin synthesis was up-regulated in *Artemisia annua* under MeJA treatment, resulting in the increase in artemisinin content [57]. Chen et al. [58] found by combined metabolomic and transcriptomic analysis that MeJA promotes the expression of key genes for flavonoid synthesis in safflower, thereby increasing the flavonoid content. 

MeJA enters the plant and is metabolized to JA-IIe, thus activating downstream gene expression to function [59]. Although *JAR1* can conjugate JA with euceucine to form JA-IIE, we found that *JAR1* expression levels were significantly up-regulated in *S. tenuifolia* under the action of MeJA through transcriptomic data, suggesting that *JAR1* may directly mediate plant JA signaling, which is similar to the study in *Artemisia annua* [60]. When JA-IIe levels are elevated in plants, JA-IIe forms the SCF^COI1^-JA-IIe complex with *COI1*, which mediates the degradation of *JAZ* by 26S proteins, prompting the release of MYC2 transcription factors to bind to downstream functional genes, thereby initiating the expression of downstream genes. It was shown that the expression levels of key genes related to JA signaling were all up-regulated after MeJA treatment in *S. tenuifolia*, suggesting that MeJA may regulate JA signaling and promote the expression of downstream genes. 

Terpenoids, as defensive substances, can resist external biological and abiotic stresses [61,62]. The synthesis of terpenoids includes two parts: terpenoids skeleton synthesis and skeleton modification [63]. The terpenoids skeleton synthesis is realized by MVA and MEP, whereas the monoterpenoids are mainly synthesized by MEP. In this study, we found that the expression levels of key enzyme genes (*DXS*, *DXR*, *MCT*, *MCS*, and *HDS*) for skeleton synthesis were up-regulated under the action of MeJA, which is similar to the studies in *Chrysanthemum indicum* var. *aromaticum* [61], *Curcuma wenyujin* [64], and *Artemisia annua* [60], indicating that the synthesis of monoterpenoid in *S. tenuifolia* is mediated by the MEP pathway. In addition, we identified two *HDR* genes in *S. tenuifolia*, and the researchers showed that *HDR* was highly expressed in *Artemisia annua* [57] and *Salvia miltiorrhiza* [65] after MeJA treatment, but we found that the expression levels of *StHDR* after MeJA treatment were significantly down-regulated compared with the CK group, and the specific molecular mechanism needs to be further explored. The expression levels of monoterpenoid skeleton modifying enzyme genes (*GPS*, *LS*, *L3OH*, *IPR*, *PR*) of *S. tenuifolia* were significantly up-regulated after MeJA treatment, and the increase was especially significant in MJ100 treatment. Menthol is the predominant monoterpene component of *Mentha canadensis*, whereas menthone is the direct prerequisite substance for menthol. Qi et al. [66] found in their study on the influence of MeJA on the transcriptional profile of *Mentha canadensis* that MeJA prompted the upregulation of the expression of nine key enzyme genes for menthol synthesis, such as *GPS* and *LS*, which is consistent with our study in *S. tenuifolia*. 

Transcription factors play an important role in plant secondary metabolism and regulate the synthesis of plant secondary metabolites by binding to cis-acting elements of downstream genes to initiate the expression of structural genes [67,68]. In contrast, numerous studies have shown that jasmonic acid analogues (JA) can activate or repress the activity of transcription factors thereby affecting the expression of genes of key enzymes for secondary metabolite synthesis [69,70,71]. In total, 887 differentially expressed transcription factors were identified in this study, including 35 gene families such as C2H2, bHLH, MYB, WRKY and bZIP. Notably, the expression levels of most genes of the C2H2 family were significantly down-regulated by MeJA treatment, whereas the expression of most genes of other families was up-regulated. A similar phenomenon was also found in Peppermint [72]. Pauw et al. [73] found that C2H2 family *ZCT* genes act as transcriptional repressors in response to MeJA thereby regulating alkaloid biosynthesis in *Catharanthus roseus*. The bHLH is an indispensable transcription factor in biological processes, which mainly regulates the biosynthesis of plant secondary metabolites [74,75,76]. Ludwig et al. discovered the first bHLH family gene in plants, which was shown to regulate anthocyanin synthesis in maize [77]. MYC2, a member of the bHLH family, is a central regulator in JA signal transduction [78]. Studies have shown that *AsMYC2* controls the expression of *ASS1* in response to JA signaling pathway and participates in the regulation of sesquiterpene biosynthesis of *Aquilaria sinensis* [79]. Based on the transcriptomic data, we identified two MYC2 genes and found that their expression levels were significantly up-regulated after MeJA treatment. We speculate that MYC2 may mediate JA signaling and regulate the expression of downstream functional genes to influence the synthesis of monoterpenoid in *S. tenuifolia*. In addition, we found that the expression levels of WRKY, MYB, bZIP and other transcription factors changed significantly under MeJA. Most of the transcription factors function in a mutually binding manner, forming a vast network of plant gene regulation [80,81,82]. Yang et al. [70] showed that MYB21 and MYC2 interact to regulate the expression of terpenoid synthase genes in flowers of *Freesia hybrida*, which affects the biosynthesis of linalool. Therefore, the identification and study of related transcription factors will help to further explore the molecular basis of MeJA regulation of monoterpene synthesis in *S. tenuifolia*.

## 5. Conclusions

In conclusion, we found that MeJA positively regulates the increase in monoterpenoid content in *S. tenuifolia* by regulating the expression of key enzyme genes in its biosynthetic pathway. In total, 5932 genes responding to MeJA were identified; changes in the expression of these genes were detected in the leaves of *S. tenuifolia* exposed to MeJA with four MeJA concentrations (CK, MJ50, MJ100 and MJ250), respectively. These genes are involved in flavonoid synthesis, monoterpenoid synthesis, JA signaling, and transcription factors. MeJA regulates the synthesis of monoterpenoids in *S. tenuifolia* without the regulation of transcription factors. We identified a total of 887 differentially expressed transcription factors, including 35 gene families; most of them showed significantly elevated expression levels under MeJA treatment. Phenotypic and differentially expressed gene association analysis revealed that monoterpenoid biosynthesis in *S. tenuifolia* was more associated with genes involved in plant trichome branching, phytohormone signaling and transcriptional regulation. The DEGs identified in this study could be the focus of future research on the biosynthesis of monoterpenoids in *S. tenuifolia*. We report the first study of the transcriptome of *S. tenuifolia* under the action of MeJA, which will provide a preliminary theoretical basis for further investigating the molecular mechanism of MeJA regulation of secondary metabolism in *S. tenuifolia*.

## Figures and Tables

**Table 1:** Assembly of *S. tenuifolia* transcripts and unigenes.

Length Range	Transcripts	Unigenes
300–500	35,571(14.39%)	25,482(28.83%)
500–1000	48,578(19.66%)	26,415(29.89%)
1000–2000	58,610(23.72%)	15,654(17.71%)
≥2000	104,359(42.23%)	20,822(23.56%)
Total Number	247,118	88,373
N50 Length	3187	2678

## Data Availability

The datasets are publicly available at NCBI with Sequence Read Archive (SRA) accession: PRJNA908244.

## Supplementary Materials

The following supporting information can be downloaded at: https://www.mdpi.com/article/10.3390/cimb45040179/s1, Table S1: Statistics of transcriptome sequencing data of *S. tenuifolia*; Table S2: Differentially expressed gene GO function annotation; Table S3: Primers used for qRT-PCR; Figure S1: Length distribution of transcript and unigenes; Figure S2: Differentially expressed gene Venn diagram; Figure S3: A heat map depicting the overall trend of the differential expression profiles of the transcription factor genes in response to MeJA treatments.
