# Supplementary material for: Transcriptomics Reveals the Molecular Basis for Methyl Jasmonate to Promote the Synthesis of Monoterpenoids in Schizonepeta tenuifolia Briq
Source: Curr Issues Mol Biol. 2023 Mar 24;45(4):2738–56. doi: 10.3390/cimb45040179 (PMC10137224; doi:10.3390/cimb45040179)
Supplement: Supplementary file 1 [file cimb-45-00179-s001.zip › Supplementary Information.pdf]

Supplementary information

Table S1. Statistics of transcriptome sequencing data of *S. tenuifolia*.

Sample	ReadSum	BaseSum	GC%	Q20(%)	Q30(%)
CK1	24968332	7490499600	37.12	97.04	92.34
CK2	25524015	7657204500	38.97	97.34	92.96
CK3	24116574	7234972200	36.78	96.93	92.19
MJ100.1	25449027	7634708100	43.35	97.4	92.96
MJ100.2	29727880	8918364000	41.98	97.48	93.13
MJ100.3	27137753	8141325900	42.47	97.61	93.39
MJ250.1	26458433	7937529900	42.83	97.51	93.24
MJ250.2	28094194	8428258200	43.23	97.44	93.1
MJ250.3	29188328	8756498400	44.13	97.48	93.12
MJ50.1	25287775	7586332500	38.2	97.26	92.85
MJ50.2	24721460	7416438000	44.91	97.65	93.53
MJ50.3	23966258	7189877400	44.06	97.64	93.48

Table S2. Differentially expressed gene GO function annotation.

**ID**	**classification**	**GO Function**
GO:0006952	Biological Process	defense response
GO:0009768	Biological Process	photosynthesis, light harvesting in photosystem I
GO:0009809	Biological Process	lignin biosynthetic process
GO:0008152	Biological Process	metabolic process
GO:0010200	Biological Process	response to chitin
GO:0009451	Biological Process	RNA modification
GO:0010150	Biological Process	leaf senescence
GO:0016554	Biological Process	cytidine to uridine editing
GO:0043161	Biological Process	proteasome-mediated ubiquitin-dependent protein catabolic process
GO:0071483	Biological Process	cellular response to blue light
GO:0016556	Biological Process	mRNA modification
GO:0010942	Biological Process	positive regulation of cell death
GO:0034599	Biological Process	cellular response to oxidative stress
GO:0007623	Biological Process	circadian rhythm
GO:0009690	Biological Process	cytokinin metabolic process

Transfer to S2 above.

ID	classification	GO Function
GO:2000022	Biological Process	regulation of jasmonic acid mediated signaling pathway
GO:0009231	Biological Process	riboflavin biosynthetic process
GO:0015979	Biological Process	photosynthesis
GO:0006412	Biological Process	translation
GO:0010380	Biological Process	regulation of chlorophyll biosynthetic process
GO:0009637	Biological Process	response to blue light
GO:0009535	Cellular Component	chloroplast thylakoid membrane
GO:0005840	Cellular Component	ribosome
GO:0009941	Cellular Component	chloroplast envelope
GO:0046658	Cellular Component	anchored component of plasma membrane
GO:0022626	Cellular Component	cytosolic ribosome
GO:0022625	Cellular Component	cytosolic large ribosomal subunit
GO:0048046	Cellular Component	apoplast
GO:0009570	Cellular Component	chloroplast stroma
GO:0005618	Cellular Component	cell wall
GO:0010287	Cellular Component	plastoglobule
GO:0009522	Cellular Component	photosystem I
GO:0043231	Cellular Component	intracellular membrane-bounded organelle
GO:0009534	Cellular Component	chloroplast thylakoid
GO:0010445	Cellular Component	nuclear dicing body
GO:0005777	Cellular Component	peroxisome
GO:0009579	Cellular Component	thylakoid
GO:0009543	Cellular Component	chloroplast thylakoid lumen
GO:0009536	Cellular Component	plastid
GO:0003700	Molecular Function	DNA-binding transcription factor activity
GO:0020037	Molecular Function	heme binding
GO:0102336	Molecular Function	3-oxo-arachidoyl-CoA synthase activity
GO:0102337	Molecular Function	3-oxo-cerotoyl-CoA synthase activity
GO:0102338	Molecular Function	3-oxo-lignoceronyl-CoA synthase activity
GO:0043565	Molecular Function	sequence-specific DNA binding
GO:0031409	Molecular Function	pigment binding
GO:0016758	Molecular Function	transferase activity, transferring hexosyl groups
GO:0009055	Molecular Function	electron transfer activity
GO:0004714	Molecular Function	transmembrane receptor protein tyrosine kinase activity
GO:0004842	Molecular Function	ubiquitin-protein transferase activity
GO:0004427	Molecular Function	inorganic diphosphatase activity
GO:0003735	Molecular Function	structural constituent of ribosome
GO:0050404	Molecular Function	zeatin O-beta-D-xylosyltransferase activity
GO:0005506	Molecular Function	iron ion binding
GO:0004497	Molecular Function	monooxygenase activity
GO:0050662	Molecular Function	coenzyme binding

Table S3. Primers used for qRT-PCR.

Number	Gene name	Sequence (5'-3')
1	*ACT*	F: GGTGTTATGGTTGGGATGGG<br>R: GCTGGCACGTTGAAGGTCTC
2	*DXS1*	F: AAGAGGGGAGTAGAGTTGCG<br>R: TCCATCAAGAGGCTTGCAGA
3	*DXS3*	F: GGTGGTGCATGATGTGGATC<br>R: GGATCAGCTCTGCCTCATCT
4	*DXR*	F: CTCGACTGGCCAATCCCTAA<br>R: CCGCCCTTATAAACACGTGG
5	*HMGR*	F: ACAACTGATTTTCCACGCCC<br>R: TTCAAGTGGTACCGTCCTCC
6	*HMGS*	F: TCCAACTCATGCCTCGACTT<br>R: TGTCAGCCACCATGTTCTCT
7	*GPS*	F: GCTATTGTGCGAGACTGTGG<br>R: ATCATCATGGAGGAGGCTGG
8	*PR*	F: CGCATGAAGAAACTCGAGGG<br>R: TCCAACACTCTCGACACTCC
9	*JAZ3*	F: CTCCTTTCCCTGTGGTGCTA<br>R: TTTGCACTTTCCCTCGAACG

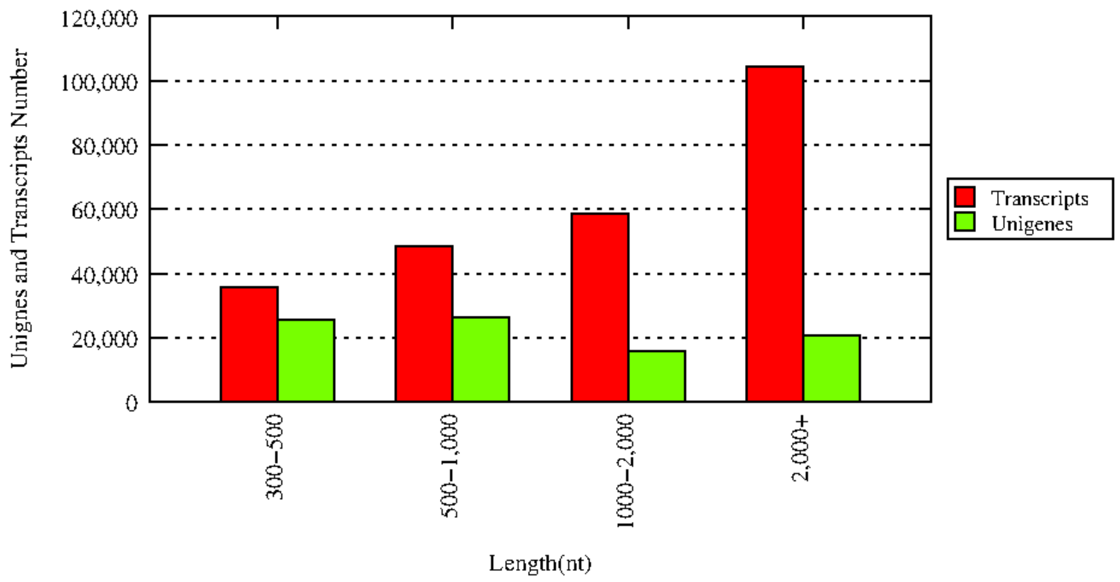

Figure S1. Length distribution of transcript and unigenes.

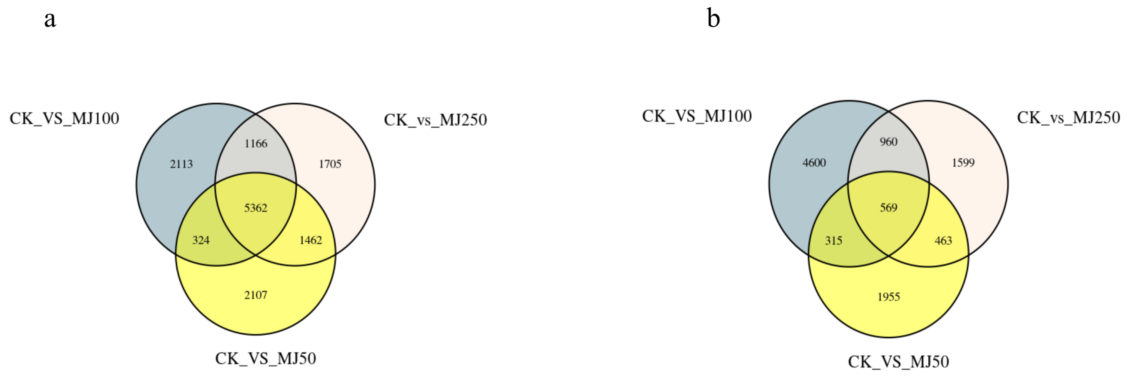

Figure S2. Differentially expressed gene Venn diagram. a Differential expression up-regulates genes; b Differential expression down-regulated genes.

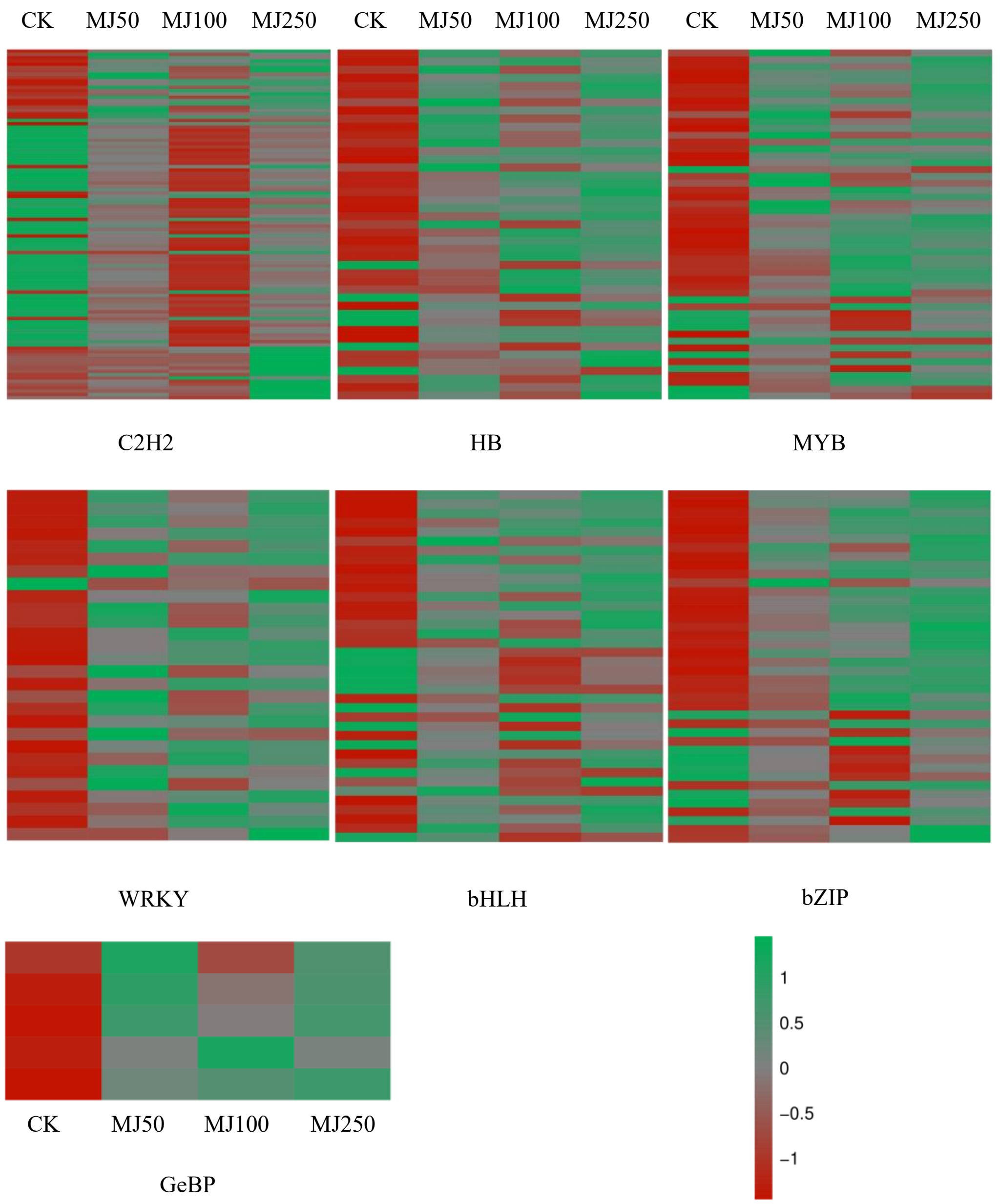

Figure S3. A heat map depicting the overall trend of the differential expression profiles of the transcription factor genes in response to MeJA treatments.
